# Cocaine-Induced Vasculitis

**DOI:** 10.5041/RMMJ.10263

**Published:** 2016-10-31

**Authors:** Mark Berman, Daphna Paran, Ori Elkayam

**Affiliations:** 1 Department of Rheumatology, Tel-Aviv Sourasky Medical Center, Tel Aviv, Israel; 2 Sackler Faculty of Medicine, Tel Aviv University, Tel Aviv, Israel

**Keywords:** Cocaine, levamisole, vasculitis

## Abstract

The use of cocaine continues to grow worldwide. One of the possible side-effects of cocaine is vasculitis. Two distinct vasculitic syndromes have been described due to cocaine. One is cocaine-induced midline destructive lesion, secondary to a direct vasoconstrictor effect of cocaine, inducing ischemic necrosis of the septal cartilage and perforation of the nasal septum, mimicking findings of granulomatosis with polyangiitis in the upper airways. The other is ANCA-associated vasculitis, attributed to the levamisole component that contaminates about 70% of the cocaine. This type of vasculitis may be myeloperoxidase (MPO) and proteinase 3 (PR3) positive, and its main manifestations are typical cutaneous findings, arthralgia, otolaryngologic involvement, and agranulocytosis. A high degree of suspicion and awareness is needed in order properly to diagnose and treat these patients.

## INTRODUCTION

The use of cocaine continues to grow worldwide, including in Israel, where it is estimated that around 1% of individuals between 18–40 years of age have used cocaine at least once in their lifetime. About 70% of cocaine is contaminated with levamisole. Two distinct vasculitic syndromes due to cocaine use have been described. The first is cocaine-induced midline destructive lesion (CIMDL), with ischemic necrosis of the septal cartilage and perforation of the nasal septum, mimicking granulomatosis with polyangiitis in the upper airways. The second is anti-neutrophil cytoplasmic antibody (ANCA)-associated vasculitis attributed to the levamisole component, myeloperoxidase (MPO) and proteinase 3 (PR3) positive, whose main manifestations are typical cutaneous findings, arthralgias, otolaryngologic involvement, and agranulocytosis.

Cocaine use and its related complications are well-known public health issues. Cocaine is commonly snorted, inhaled, or injected into the veins. It is estimated that about 1.5% of the USA population uses cocaine regularly. Israel is not infrequently listed in connection with cocaine in the World Drug Report for 2013, issued by the United Nations Office on Drugs and Crime, which discusses trends in the world.^1^ It is difficult to find up-to-date figures on the scope of cocaine trafficking at the local level in Israel. However, the latest survey (from 2009) by Israel’s Anti-Drug Authority on cocaine noted a clear trend: cocaine use had doubled by 2009 compared with 2005, and close to 1% of all Israelis aged 18–40 years indicated that they had used cocaine.

The toxic effects of cocaine include possibly irreversible structural changes of the brain, heart, lung, and other organs, such as the liver and kidney.^2^ A 2009 USA national survey found that ~70% of cocaine is contaminated by levamisole.^3^,^4^ Levamisole, a medication used to treat parasitic worm infection, is added to cocaine because it potentiates its stimulant effects by inhibiting both monoamine oxidase and catechol-O-methyltransferase activity, thereby prolonging the action of catecholamines in the neuronal synapse and increasing the reuptake inhibition. In addition, levamisole reacts like cocaine in the “bleach test,” a quick, widely utilized street test for cocaine purity. Therefore, it adds weight to illicit cocaine without reducing the native drug’s apparent purity, as occurs with other bulking agents such as sugar or lidocaine.^4^

Originally marketed as an anti-helminthic agent, levamisole was also found to have major immunomodulatory properties, and it was therefore used to treat colon cancer, rheumatoid arthritis, and pediatric nephrotic syndrome. Reports of neutropenia led to its withdrawal from the USA market in 1999, although it is still used as a deworming agent in veterinary medicine, and it is still marketed as an anti-helminthic and immunomodulatory agent in some countries.

Importantly, some of the toxic effects of cocaine are attributable to levamisole. There is growing recognition of levamisole-induced agranulocytosis, vasculitis, midline destructive lesions, and other complications in cocaine users. One of the less known effects of cocaine use is its ability to induce several types of vasculitis, especially those that mimic ANCA-associated vasculitis. Two types of cocaine-induced syndromes have been described over the past two decades: one is directly related to the local effects of snorting cocaine that may lead to midline destructive lesions and therefore mimics vasculitis lesions found in granulomatosis with polyangiitis (GPA), and the other is attributed to levamisole and behaves like drug-induced ANCA-associated vasculitis.

## COCAINE-INDUCED MIDLINE DESTRUCTIVE LESIONS

Nasal insufflation of cocaine may cause lesions in the mucosa. Progressive damage of the mucosa and perichondrium associated with chronic cocaine use leads to ischemic necrosis of the septal cartilage and perforation of the nasal septum. The mucosal damage induced by cocaine is multifactorial, with the vasoconstrictive effect of the drug thought to be the most important factor.^5^–^7^ However, the irritant effect of the adulterants of the drug, the traumatic effect on the mucosa caused by cocaine crystals insufflated at high velocity, and the recurrent nasal infections all seem to contribute to chronic tissue destruction.^6^,^8^ Cocaine-induced lesions occasionally cause extensive destruction of the osteocartilaginous structures of the nose, sinuses, and palate that mimics the clinical picture of other diseases associated with necrotizing midfacial lesions.^7^ Progressive nasal obstruction, epistaxis with crusting, and ulceration of the nasal mucosa with or without septal perforation are also characteristic manifestations of nasal involvement by GPA. The differentiation between CIMDLs and limited GPA may be difficult, particularly if the patients do not mention their substance abuse.

Anti-neutrophil cytoplasmic antibodies directed against PR3 or MPO are sensitive and specific markers for the idiopathic small-vessel vasculitides, including GPA. The presence of a positive ANCA test result with either of those two markers points to the differential diagnosis of GPA. However, positive ANCA test results were found in an unexpectedly large proportion of cocaine-abusing patients with CIMDL whose lesions were clinically indistinguishable from GPA limited to the upper respiratory tract in several cases.^9^ Trimarchi et al.^7^ compared the clinical, serologic, radiographic, and histopathologic features of 18 consecutive patients who presented with CIMDL with those of 21 patients with GPA with nasal involvement who were being evaluated during the same time period. Routine ANCA tests were positive in 13 of the 18 CIMDL patients compared with 19 of 21 GPA patients. Clinical and radiographic evaluations revealed that destruction of facial midline structures was significantly more severe in CIMDL than in GPA. Although biopsies with non-specific changes were more frequent in CIMDL, and leukocytoclastic vasculitis and fibrinoid necrosis were more frequent in GPA, both were reported in the two pathologies and therefore did not contribute to the diagnosis of individual patients. In contrast to GPA, there was no other organ involvement and no significant laboratory abnormalities indicating systemic inflammation in CIMDL. Detailed analysis of the ANCAs found in the CIMDL and GPA patients showed that none of the eight perinuclear ANCAs in CIMDL patients reacted with MPO, while four reacted with PR3, three with human neutrophil elastase (HNE), and two had double positivity to PR3 and HNE. All of the five cytoplasmatic ANCAs reacted with PR3, and two of them also reacted with HNE. In contrast, 18 of the 19 ANCA-positive GPA patients displayed concurrent p-/MPO-ANCA or c-/PR3-ANCA reactivity.

It is known that HNE and PR3 belong to the same family of serine proteases. Wiesner et al.^10^ reported an unexpectedly high frequency (84%) of HNE ANCAs in patients presenting with CIMDL. In contrast, no HNE ANCAs were detected in their patients with GPA or microscopic polyangiitis, and HNE ANCAs were detected only rarely in patients with other autoimmune diseases or vasculitis. Many of the sera obtained from patients with CIMDL also reacted with PR3. Consequently, those authors concluded that HNE ANCAs occurring in patients with midline destructive lesions may discriminate between CIMDL and GPA.^7^,^10^

## LEVAMISOLE-INDUCED VASCULITIS

As noted before, approximately 70% of cocaine in the USA is contaminated with levamisole. A growing number of reports describing levamisole-induced vasculitis have appeared during the last 20 years. The first report was published as a case series in 1999.^11^ Five children who were treated with levamisole for nephrotic syndrome for an average of 24 months developed purpuric and erythematous macules, rapidly enlarging necrotic areas, purpuric papules/plaques, and hemorrhagic bullae. Skin involvement of the external ears was present in all patients. Subsequent case studies with a similar profile of skin findings confirmed this association. The lesions usually were resolved by 2–3 weeks after drug discontinuation.

The first report of vasculitis induced by levamisole-contaminated cocaine was published in 2010, and the condition was characterized by typical cutaneous findings, agranulocytosis/neutropenia, and a positive ANCA.^12^ Several similar case reports later appeared in the literature.

McGrath et al.^13^ described ANCA-positivity associated with levamisole-contaminated cocaine. In their study, 327 new ANCA-positive patients during 2009–2010 were identified and reviewed. Active cocaine use was identified in 30 cases. The medical records of 18 active cocaine users were available for review: 16 had skin manifestations consisting of necrotic lesions (*n*=3), purpura (*n*=6), digital abscesses (*n*=1), ecchymotic bullous skin lesions (*n*=1), and purpuric lesions over earlobes (*n*=5) as described in classic levamisole-induced vasculitis. Arthralgias were reported in 83%, which generally involved the large joints. A total of 72% of patients reported at least one constitutional symptom such as fever, night sweats, weight loss, malaise, or myalgia. Otolaryngologic involvement was present in 44% of cases, most commonly sinusitis and recurrent rhinorrhea. Twenty-eight percent of patients had leucopenia at presentation; four patients had an absolute neutrophil count below 100, and one had profound leucopenia requiring granulocyte macrophage colony-stimulating factor. Abnormal urinalysis was present in eight patients at diagnosis, and two of them developed severe acute renal failure. One patient underwent kidney biopsy revealing pauci-immune focal necrotizing and crescentic glomerulonephritis. Pulmonary hemorrhage, not requiring intubation, was reported in three patients; none of them had impairment of renal function. Overall, there was no definitive evidence of pulmonary–renal syndrome in this cohort.^13^

On biopsy, skin lesions may show thrombotic vasculopathy, vasculitis, or a combination of the two. The vasculitis is believed to be immune-mediated based on the presence IgM, IgG, IgA, and C3 complexes. There are very few reports of biopsies of internal organs: one renal biopsy in a patient with acute kidney injury revealed pauci-immune focal necrotizing and crescentic glomerulonephritis.^14^–^16^

Levamisole-induced vasculitis has unusual auto-antibody findings. High-titer perinuclear ANCAs are almost always present (86%–100%), and about 50% of the cases also have cytoplasmic ANCAs.^13^ However, the specific antigens responsible for generating these positive ANCA fluorescent patterns are not yet clearly defined. Antibodies against myeloperoxidase (anti-MPO), the antibody most often responsible for a perinuclear ANCA pattern, are found in almost every case. Importantly, anti-MPO titers in cocaine-associated ANCAs may be very high, up to 15-fold higher than in patients with idiopathic ANCA-associated vasculitis.^13^ Antibodies against PR3, the autoantibody most commonly associated with a cytoplasmic ANCA pattern, are present in about 50% of these patients, but they may be directed against HNE because of cross-reactivity, as noted earlier in connection with CIMDL.

Levamisole-induced vasculitis remains a challenging diagnosis. It may not be initially suspected if patients are not queried about illicit drug use or if they deny drug use when questioned. It is possible to perform a urine drug toxicology screen because cocaine remains in the urine for 48 to 72 hours following use. A definitive connection to levamisole may, however, be difficult to establish even with a positive cocaine screen because levamisole is rapidly absorbed and has a short half-life (5.5–6 hours). A recent study demonstrated that over two-thirds of urine samples positive for cocaine had detectable levels of levamisole on gas chromatography/mass spectroscopy.^17^ That study was carried out in an inner-city emergency department, and it emphasized the need for a very high degree of clinical suspicion early in disease presentation in order definitively to confirm exposure.

Management of levamisole-induced vasculitis is supportive. The cornerstone of treatment is halting further exposure to cocaine. Medical treatment may include wound dressing and antibiotics in super-imposed infections. Some untreated cutaneous lesions may regress in a few weeks after stopping cocaine use.^18^ There is currently no evidence that systemic corticosteroids modify the clinical course, but they may be reserved for cases that are unresponsive to supportive therapy alone, e.g. debilitating arthropathy, strikingly elevated C-reactive protein levels, or biopsy-proven vasculitis.^19^ Non-compliance with therapy and ongoing cocaine use make this a challenging patient group to manage.

## CONCLUSIONS

Cocaine abuse is an expanding public health concern. Cocaine-induced vasculitis may present in different forms. Snorting cocaine may induce midline destructive lesions which are often ANCA-positive and which may be indistinguishable from otolaryngologic lesions of GPA. Aggressive local midline destruction, lack of systemic symptoms, and HNE ANCA-associated antibodies may point toward a cocaine-induced lesion. The other cocaine-induced vasculitis phenotype, systemic ANCA-associated vasculitis, is due to the levamisole component of the drug. It is characterized by typical hemorrhagic skin lesions, leucopenia, rare renal involvement, and very high titers of perinuclear and cytoplasmic ANCA-associated autoantibodies. Cessation of cocaine use is essential and may be the only step required to resolve this clinical condition. In view of the increasing use of cocaine, a high degree of suspicion and awareness is needed in order properly to diagnose and treat patients with cocaine-induced vasculitis.

## Abbreviations

ANCA: anti-neutrophil cytoplasmic antibody
CIMDL: cocaine-induced midline destructive lesion
GPA: granulomatosis with polyangiitis
HNE: human neutrophil elastase
MPO: myeloperoxidase
PR3: proteinase 3
